# Treatment plan comparison between Tri-Co-60 magnetic-resonance image-guided radiation therapy and volumetric modulated arc therapy for prostate cancer

**DOI:** 10.18632/oncotarget.20039

**Published:** 2017-08-08

**Authors:** Jong Min Park, So-Yeon Park, Chang Heon Choi, Minsoo Chun, Jin Ho Kim, Jung-In Kim

**Affiliations:** ^1^ Department of Radiation Oncology, Seoul National University Hospital, Seoul, Republic of Korea; ^2^ Institute of Radiation Medicine, Seoul National University Medical Research Center, Seoul, Republic of Korea; ^3^ Biomedical Research Institute, Seoul National University College of Medicine, Seoul, Republic of Korea; ^4^ Robotics Research Laboratory for Extreme Environments, Advanced Institutes of Convergence Technology, Suwon, Republic of Korea

**Keywords:** prostate cancer, magnetic-resonance image-guided radiation therapy, volumetric modulated arc therapy

## Abstract

To investigate the plan quality of tri-Co-60 intensity-modulated radiation therapy (IMRT) with magnetic-resonance image-guided radiation therapy compared with volumetric-modulated arc therapy (VMAT) for prostate cancer. Twenty patients with intermediate-risk prostate cancer, who received radical VMAT were selected. Additional tri-Co-60 IMRT plans were generated for each patient. Both primary and boost plans were generated with tri-Co-60 IMRT and VMAT techniques. The prescription doses of the primary and boost plans were 50.4 Gy and 30.6 Gy, respectively. The primary and boost planning target volumes (PTVs) of the tri-Co-60 IMRT were generated with 3 mm margins from the primary clinical target volume (CTV, prostate + seminal vesicle) and a boost CTV (prostate), respectively. VMAT had a primary planning target volume (primary CTV + 1 cm or 2 cm margins) and a boost PTV (boost CTV + 0.7 cm margins), respectively. For both tri-Co-60 IMRT and VMAT, all the primary and boost plans were generated that 95% of the target volumes would be covered by the 100% of the prescription doses. Sum plans were generated by summation of primary and boost plans. In sum plans, the average values of V70 Gy of the bladder of tri-Co-60 IMRT vs. VMAT were 4.0% ± 3.1% vs. 10.9% ± 6.7%, (*p* < 0.001). Average values of V70 Gy of the rectum of tri-Co-60 IMRT vs. VMAT were 5.2% ± 1.8% vs. 19.1% ± 4.0% (*p* < 0.001). The doses of tri-Co-60 IMRT delivered to the bladder and rectum were smaller than those of VMAT while maintaining identical target coverage in both plans.

## INTRODUCTION

Intensity-modulated radiation therapy (IMRT) and volumetric modulated arc therapy (VMAT) are known to be effective for the treatment of prostate cancer [1–3]. To perform IMRT or VMAT for prostate cancer, considerable margins are generally applied for the generation of the planning target volume (PTV) since the internal motion of the prostate is known to be large (up to 1.2 cm and 1.5 cm for intrafractional and interfractional motions, respectively) [4, 5]. These large PTV margins generally cause overlapping between the target volumes and the neighboring organs at risk (OARs). To deliver prescription doses to the PTVs, these neighboring organs sometimes irradiated by high doses. This occasionally results in complications such as rectal bleeding, despite the superior ability of IMRT and VMAT to generate rapid dose fall-off around the target volume [6–8]. Adaptive radiation therapy (ART) can reduce the PTV margins effectively; however, there are a number of practical obstacles to performing ART routinely in the clinic, such as a considerable increase in patient imaging dose and complicated procedures of ART [9–11].

Recently, a magnetic-resonance image-guided radiation therapy (MR-IGRT) system was introduced in the field of radiation therapy (the ViewRay™ system, ViewRay Inc., Cleveland, OH, USA) [12, 13]. This MR-IGRT machine can perform ART with daily volumetric MR images combined with rapid optimization and dose calculation algorithms [12, 13]. In addition, the ViewRay system can perform respiratory gating with near-real-time cine sagittal MR images during treatment [14]. This facilitates the monitoring of movements of the target volume during treatment, which enables a reduction of the PTV margins. In this respect, this system enables the generation of more favorable treatment plans compared with the conventional radiation therapy technique. However, the beam delivery system of the ViewRay system is relatively inferior to that of the conventional linac, which potentially decreases the plan quality [14, 15]. To be compatible with the MR imaging system, the ViewRay system adopted Co-60 sources as the beam source, which could result in larger penumbrae and lower penetrating power than those of the linac [14, 15]. In addition, the multi-leaf collimator (MLC) leaf width of the ViewRay system is larger than those of conventional linacs, which is 1.05 cm at the isoplane located at source to surface distance of 105 cm, which could potentially degrade the plan quality [14]. In conclusion, the imaging capability of the ViewRay system could improve the plan quality; however, its beam delivery system also has the potential to degrade it.

Several studies have investigated the quality of the tri-Co-60 IMRT plans that were generated with the ViewRay system for various treatment sites [14–20]. Wooten *et al.* demonstrated the comparable plan quality of the tri-Co-60 IMRT to that of linac-based IMRT for diseases in the abdominal, pelvic, thorax and head and neck regions [16]. Kishan *et al.* also demonstrated a slightly inferior yet comparable plan quality of the tri-Co-60 IMRT with that of linac-based VMAT for liver stereotactic ablative radiotherapy (SABR) [18]. Merna *et al.* showed that the tri-Co-60 IMRT plans for lung SABR were comparable to those of linac-based IMRT when the target volumes were large and located at the central regions of the lung [17]. Park *et al.* performed a planning study for lung SABR with small target volumes involving use of the ViewRay system compared with the VMAT [14]. In that particular case, the target conformity of the tri-Co-60 IMRT plans became inferior to that of the VMAT plans as the target volume sizes decreased due to the large MLC leaf width. Choi *et al.* reported an inferior plan quality with the tri-Co-60 IMRT compared with that of linac-based VMAT for spine SABR because of the proximity of the target volume to the spinal cord [15]. Various planning studies have investigated the plan quality of the tri-Co-60 IMRT; however, no thorough study has been performed for prostate cancer [14–20]. The study performed by Wooten *et al.* included a few prostate cancer cases; however, the number of cases were limited and the target volumes of the tri-Co-60 IMRT and the linac-based IMRT were identical to each other [16]. Therefore, the quality of the tri-Co-60 IMRT plans with MR-IGRT considering the target margin reduction capability is unclear. In this study, we investigated the plan quality of the tri-Co-60 IMRT with reduced target margins from the clinical target volumes (CTVs) in comparison with that of VMAT with conventional target margins to generate the PTVs. By retrospectively selecting patients with intermediate risk prostate cancer, who received radical VMAT, we additionally generated the tri-Co-60 IMRT plans and compared those to the VMAT plans.

## RESULTS

**Figure 1:**
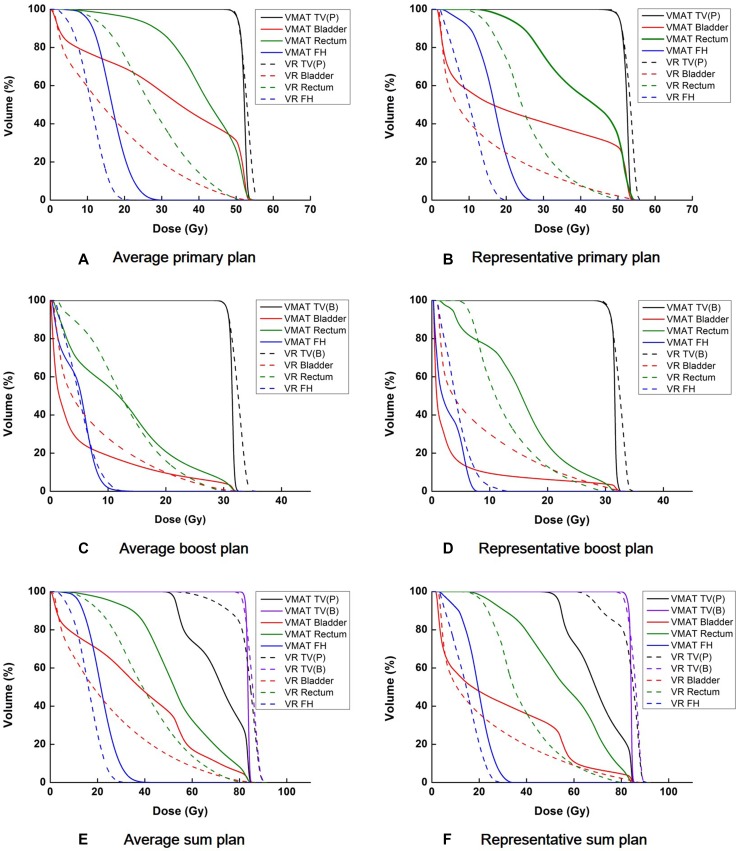
Average dose volume histograms (DVHs) and DVHs of a representative patient (patient 18) are shown for the target volume, bladder, rectum, and femoral heads The average DVHs from a primary plan (**A**), boost plan (**C**), and sum plan (**E**) are shown. The DVHs of a representative patient, from a primary plan (**B**), boost plan (**D**), and sum plan (**F**) are also shown. The DVHs of the volumetric modulated arc therapy (VMAT) plans are shown with solid lines while those of the tri-Co-60 intensity modulated radiation therapy (IMRT) plans are shown with dashed lines. TV(P), TV(B), FH, and VR are abbreviations of primary target volume, boost target volume, femoral heads and the ViewRay system, respectively.

The dose-volumetric parameters of the primary and boost plans are shown in Table 1. Those of the sum plans are shown in Table 2. Average dose volume histograms (DVHs) and DVHs of a representative patient case (patient 18) for the primary, boost and sum plans are shown in Figure 1.

**Table 1:** Dose-volumetric parameters and beam-on times of the primary and boost plans for prostate cancer

	VMAT	Tri-Co-60 IMRT	*p*
Primary PTV of the primary plan			
Volume (cc)	320.2 ± 40.5	45.7 ± 13.2	< 0.001
D_1%_ (Gy)	53.5 ± 0.4	55.4 ± 0.4	< 0.001
D_2%_ (Gy)	53.3 ± 0.3	55.2 ± 0.4	< 0.001
D_98%_ (Gy)	49.6 ± 0.2	49.8 ± 0.2	< 0.001
D_99%_ (Gy)	49.0 ± 0.3	49.5 ± 0.3	< 0.001
Maximum dose (Gy)	54.5 ± 0.6	56.0 ± 0.4	< 0.001
Mean dose (Gy)	52.1 ± 0.2	52.8 ± 0.3	< 0.001
Minimum dose (Gy)	44.6 ± 1.3	46.3 ± 0.9	< 0.001
Conformity index	1.00 ± 0.01	1.10 ± 0.08	< 0.001
Homogeneity index	0.07 ± 0.01	0.10 ± 0.01	< 0.001
Gradient index	3.41 ± 0.13	8.29 ± 1.31	< 0.001
Beam-on time (min)	1.98 ± 0.01	4.60 ± 0.54	< 0.001

Boost PTV of the boost plan			
Volume (cc)	79.7 ± 20.7	36.1 ± 11.5	< 0.001
D_1%_ (Gy)	32.1 ± 0.2	34.3 ± 0.3	< 0.001
D_2%_ (Gy)	32.0 ± 0.2	34.2 ± 0.3	< 0.001
D_98%_ (Gy)	30.2 ± 0.1	30.2 ± 0.2	0.826
D_99%_ (Gy)	29.9 ± 0.2	29.9 ± 0.3	0.371
Maximum dose (Gy)	32.8 ± 0.2	34.8 ± 0.3	< 0.001
Mean dose (Gy)	31.4 ± 0.1	32.4 ± 0.2	< 0.001
Minimum dose (Gy)	27.3 ± 0.5	28.1 ± 0.7	0.001
Conformity index	0.98 ± 0.02	1.06 ± 0.07	< 0.001
Homogeneity index	0.06 ± 0.01	0.12 ± 0.01	< 0.001
Gradient index	3.59 ± 0.20	8.30 ± 1.42	< 0.001
Beam-on time (min)	1.98 ± 0.01	4.18 ± 0.67	< 0.001

*Abbreviations*: VMAT, volumetric modulated arc therapy; Tri-Co-60 IMRT, intensity modulated radiation therapy with Co-60 sources; PTV, planning target volume; D_*n*%_, the highest dose received by at least *n*% volume of a structure.

**Table 2:** Dose-volumetric parameters of sum plans for prostate cancer

	VMAT	Tri-Co-60 IMRT	*p*
Primary PTV			
D_1%_ (Gy)	84.3 ± 0.3	89.2 ± 0.6	< 0.001
D_2%_ (Gy)	84.2 ± 0.3	89.0 ± 0.6	< 0.001
D_95%_ (Gy)	53.4 ± 0.9	69.6 ± 5.8	< 0.001
D_98%_ (Gy)	52.1 ± 0.8	65.8 ± 5.5	< 0.001
D_99%_ (Gy)	51.2 ± 0.7	64.0 ± 5.3	< 0.001
Maximum dose (Gy)	85.4 ± 0.5	90.3 ± 0.7	< 0.001
Mean dose (Gy)	70.8 ± 2.5	83.0 ± 1.1	< 0.001
Minimum dose (Gy)	45.8 ± 1.6	56.8 ± 4.9	< 0.001
Conformity index	1.50 ± 0.05	4.64 ± 0.75	< 0.001

Boost PTV			
D_1%_ (Gy)	84.6 ± 0.3	89.3 ± 0.6	< 0.001
D_2%_ (Gy)	84.5 ± 0.3	89.1 ± 0.6	< 0.001
D_95%_ (Gy)	82.5 ± 0.3	81.3 ± 0.1	< 0.001
D_98%_ (Gy)	82.0 ± 0.3	80.4 ± 0.4	< 0.001
D_99%_ (Gy)	81.6 ± 0.4	79.8 ± 0.6	< 0.001
Maximum dose (Gy)	85.3 ± 0.4	90.3 ± 0.7	< 0.001
Mean dose (Gy)	83.5 ± 0.3	85.4 ± 0.5	< 0.001
Minimum dose (Gy)	78.9 ± 0.8	75.9 ± 1.7	< 0.001
Conformity index	1.22 ± 0.05	1.13 ± 0.07	< 0.001
Homogeneity index	0.03 ± 0.00	0.10 ± 0.01	< 0.001

Bladder			
Maximum dose (Gy)	85.0 ± 0.4	86.4 ± 2.4	0.021
D_55%_ (Gy)	29.5 ± 17.0	15.9 ± 10.1	< 0.001
D_30%_ (Gy)	48.0 ± 13.9	29.9 ± 12.4	< 0.001
D_25%_ (Gy)	52.0 ± 12.7	34.3 ± 12.5	< 0.001
V_80Gy_ (%)	5.5 ± 3.7	0.9 ± 1.2	< 0.001
V_75Gy_ (%)	7.9 ± 5.0	2.3 ± 2.1	< 0.001
V_70Gy_ (%)	10.9 ± 6.7	4.0 ± 3.1	< 0.001
V_65Gy_ (%)	13.9 ± 8.2	6.0 ± 4.2	< 0.001

Rectum			
Maximum dose (Gy)	84.9 ± 0.8	83.2 ± 1.9	0.002
D_50%_ (Gy)	52.5 ± 2.4	39.3 ± 4.8	< 0.001
D_20%_ (Gy)	68.9 ± 3.4	54.3 ± 4.4	< 0.001
V_80Gy_ (%)	8.2 ± 2.8	0.3 ± 0.4	< 0.001
V_75Gy_ (%)	13.4 ± 3.4	2.2 ± 0.9	< 0.001
V_70Gy_ (%)	19.1 ± 4.0	5.2 ± 1.8	< 0.001
V_65Gy_ (%)	25.6 ± 5.4	9.0 ± 3.0	< 0.001

Femoral heads			
D_5%_ (Gy)	30.5 ± 3.6	24.1 ± 2.3	< 0.001
Maximum dose (Gy)	37.0 ± 4.4	29.9 ± 3.1	< 0.001

*Abbreviations:* VMAT, volumetric Modulated Arc Therapy; Tri-Co-60 IMRT, intensity modulated radiation therapy with Co-60 sources; PTV, planning target volume; D_*n*%_, the highest dose received by at least *n*% volume of a structure; V_nGy_, percent volume receiving n Gy.

### Dose-volumetric parameters for the target volumes calculated from each of the primary and boost plans

For the primary plans, the volume of the primary PTV for VMAT was 7 times larger than that of the primary PTV for tri-Co-60 IMRT on average (320.2 ± 40.5 cc vs. 45.7 ± 13.2 cc with *p* < 0.001). The average values of the highest dose received by at least 1% of the target volume (D_1%_), and similarly D_2%_, D_98%_ and D_99%_ of the tri-Co-60 IMRT plans were consistently higher than those of the VMAT plans (all *p* < 0.001). Similarly, the maximum, minimum and mean dose to the target volume of the tri-Co-60 IMRT plans were higher compared with those of the VMAT plans (all *p* < 0.001). The target conformity, dose homogeneity, and the capability to generate a steep dose gradient around the target volume of the tri-Co-60 IMRT plans were inferior compared with those of the VMAT plans (all *p* < 0.001).

For the boost plans, similar results were observed as those of the primary plans. The average target volume of the VMAT plans was 79.7 ± 20.7 cc, while that of the tri-Co-60 IMRT plans was 36.1 ± 11.5 cc. The values of the D_1%_, D_2%_, the maximum dose, the mean dose and the minimum dose to the target volume of the tri-Co-60 IMRT plans were higher than those of the VMAT plans (all *p* ≤ 0.001). The target conformity, dose homogeneity and the capability to generate a steep dose gradient near the target volume of the tri-Co-60 IMRT plans were inferior to those of the VMAT plans (all *p* < 0.001).

### Dose-volumetric parameters for the target volumes calculated from sum plans

For the primary target volume, the average values of D_1%_, D_2%_, D_95%_, D_98%_, D_99%_, the maximum dose, the mean dose and the minimum dose of the tri-Co-60 IMRT plans were consistently higher compared with those of the VMAT plans (all *p* < 0.001). The target conformity of the VMAT was superior to that of the tri-Co-60 IMRT on average (*p* < 0.001).

With regard to the boost target volume, the average values of the D_1%_, D_2%_, the mean dose and the maximum dose were higher in the tri-Co-60 IMRT plans compared with the VMAT plans, while the average values of the D_95%_, D_98%_, D_99%_ and the minimum dose were lower in the tri-Co-60 IMRT plans than in the VMAT plans (all *p* < 0.001). This indicated wider ranges of the delivered doses inside the boost target volumes of the tri-Co-60 IMRT plans compared with the VMAT plans. Consequently, the average values of the *homogeneity index* (*HI*) also indicated worse dose homogeneity with the tri-Co-60 IMRT plans compared with the VMAT plans (*p* < 0.001). The target conformity of the tri-Co-60 IMRT plans was superior to that of the VMAT plans (*p* < 0.001).

### Dose-volumetric parameters for OARs calculated from sum plans

For bladder, the average values of D_55%_, D_30%_, D_25%_, the percent volumes of the bladder that received at least 80 Gy (V_80Gy_), V_75Gy_, V_70Gy_, and V_65Gy_ indicated that both the tri-Co-60 IMRT and VMAT plans were clinically acceptable [21]. However, the values were lower in the tri-Co-60 IMRT plans compared with the VMAT plans (all *p* < 0.001). With regard to the rectum, every dose-volumetric parameters of the rectum also showed significantly lower irradiation of the rectum in the tri-Co-60 IMRT plans compared with the VMAT plans (all *p* < 0.001). For femoral heads, the average values of D_5%_ and the maximum dose of the tri-Co-60 IMRT plans were lower compared with those of the VMAT plans (both *p* < 0.001); however, every VMAT plan remained clinically acceptable [21].

### Normal tissue irradiation and the beam-on time

The average values of the *gradient indices* (*GI*s), the percent volume that received at least 100% of the prescribed dose (V_100%_), V_90%_, V_70%_, V_50%_, V_30%_ and V_10%_ of the body structures excluding the target volumes (*body-PTV*) and the values of the entire body structure, including the target volumes, are shown in Table 3. With respect to the *body-PTV*, the average values of V_100%_, V_90%_ and V_70%_ were higher in the tri-Co-60 IMRT plans compared with the VMAT plans, while the average values of V_50%_, V_30%_ and V_10%_ were higher in the VMAT plans than in the tri-Co-60 IMRT plans (all *p* ≤ 0.002).

**Table 3:** Normal tissue irradiation of sum plans for prostate cancer

	VMAT	Tri-Co-60 IMRT	*p*
Body – PTV			
V_100%_ (cc)	0.1 ± 0.2	3.1 ± 2.9	< 0.001
V_90%_ (cc)	2.2 ± 2.0	29.3 ± 5.7	< 0.001
V_70%_ (cc)	87.0 ± 14.5	109.1 ± 18.2	< 0.001
V_50%_ (cc)	391.6 ± 50.9	290.3 ± 56.2	< 0.001
V_30%_ (cc)	1323.0 ± 163.6	1067.6 ± 179.5	< 0.001
V_10%_ (cc)	3884.6 ± 418.2	3597.0 ± 452.3	0.002

Body			
V_100%_ (cc)	96.6 ± 23.7	40.3 ± 12.4	< 0.001
V_90%_ (cc)	158.9 ± 34.3	71.6 ± 17.6	< 0.001
V_70%_ (cc)	340.8 ± 55.4	154.6 ± 30.6	< 0.001
V_50%_ (cc)	711.7 ± 89.2	335.9 ± 67.4	< 0.001
V_30%_ (cc)	1643.1 ± 197.7	1113.2 ± 191.6	< 0.001
V_10%_ (cc)	4204.8 ± 436.6	3642.7 ± 460.8	< 0.001
Gradient index (81 Gy)	7.76 ± 1.91	8.76 ± 1.72	0.007
Gradient index (50.4 Gy)	3.24 ± 0.15	5.09 ± 0.30	< 0.001

*Abbreviations:* VMAT, volumetric modulated arc therapy; Tri-Co-60 IMRT, intensity modulated radiation therapy with Co-60 sources; PTV, planning target volune; Body – PTV, body structure excluding the PTVs; V_*n*%_, percent volume receiving *n*% of the prescription dose; Body, body structure including the PTVs.

For the entire body, every value of V_100%_, V_90%_, V_70%_, V_50%_, V_30%_ and V_10%_ of the VMAT plans was consistently higher compared with those of the tri-Co-60 IMRT plans (all *p* < 0.001). The average values of *GI* with 81 Gy and 50.4 Gy indicated more rapid dose fall-off around the target volumes in the VMAT plans than in the tri-Co-60 IMRT plans [22].

## DISCUSSION

The MR-IGRT can eliminate or minimize the PTV margins without an additional imaging dose to the patient [4, 13, 14, 23, 24]. In particular, this is considerably beneficial for the administration of radiation therapy to patients with prostate cancer because generally in such cases relatively large PTV margins occur because of large internal organ movement [4, 23]. Therefore, we compared the plan quality of the MR image-guided tri-Co-60 IMRT plans with PTV margins 3 mm to that of VMAT plans with PTV margins of 7 mm (boost PTV margin), 1 cm and 2 cm (primary PTV margin). We demonstrated the potential for significantly lower doses to be delivered to the rectum and bladder in the tri-Co-60 IMRT plans compared with the VMAT plans.

The previous studies showed the tri-Co-60 IMRT plans were inferior to the VMAT plans for lung SABR and spine SABR [14, 15]. On the contrary, we observed a much improved plan quality with the ViewRay system in terms of OAR sparing compared with the use of linac-based VMAT for prostate cancer in the present study. As the target volume sizes for the prostate tumors were much larger than those of lung SABR reported in the previous study (320.0 ± 40.5 cc vs. 27.2 ± 23.5 cc), severe degradation of the target conformity was not observed in the present study [14].

**Figure 2:**
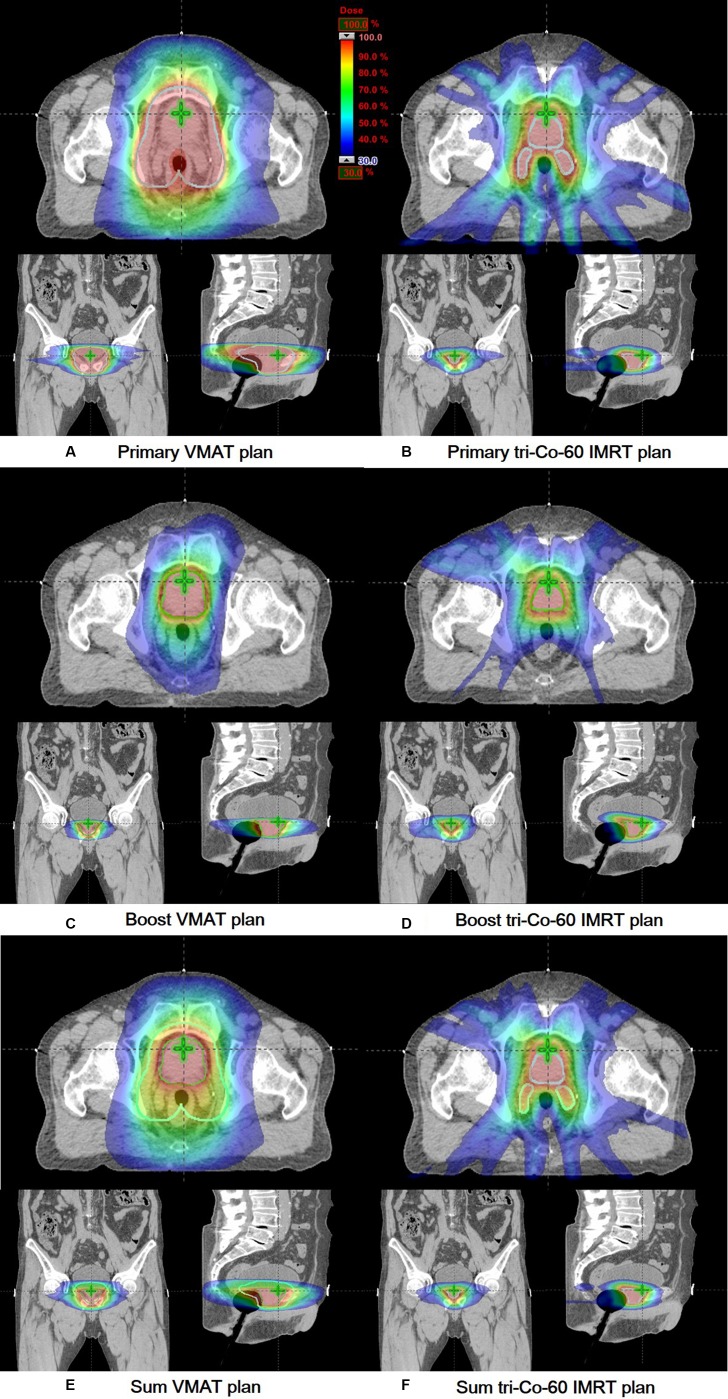
Dose distributions of a representative patient (patient 18) are shown in axial, coronal and sagittal views Dose distributions of a primary plan (**A**), boost plan (**C**), and sum plan (**E**) of the volumetric modulated arc therapy (VMAT) are shown. Those of a primary plan (**B**), boost plan (**D**), and sum plan (**F**) of the tri-Co-60 intensity-modulated radiation therapy (IMRT) are shown. The primary target volumes and the boost target volumes are delineated with cyan and green colors, respectively.

The improvement of the plan quality with the ViewRay system appears to occur because of its margin reduction capability. The margin reduction using this system resulted in the minimal overlapping between the target volumes and nearby OARs; therefore, the irradiation of OARs by high doses (in particular to the rectum and bladder) could be reduced significantly in the tri-Co-60 IMRT plans. Comparing the DVHs of the primary plans and the boost plans (Figure 1), the degree of reduction in the doses to OARs using the ViewRay system was more noticeable in the primary plans than in the boost plans. This appeared to be a result of the greater margins for the primary PTVs compared with those of the boost PTVs. The average size of the primary PTV of the VMAT was on average 7 times larger than that of the tri-Co-60 IMRT, while the average boost PTV size of the VMAT was 2.2 times larger than that of the tri-Co-60 IMRT. The considerable overlap between the primary target volume and the rectum, as well as with the bladder in the VMAT plan, is given in Figure 2. Therefore, the large penumbrae of the Co-60 sources appeared to be surmounted by the margin reduction capability of the MR images. This can also be identified with the values V_n%_ of the entire body and the values of *GI* in Table 3. If the target volumes between the tri-Co-60 IMRT and VMAT plans were identical, the value of *GI* indicates the degree of normal tissue sparing; however, the target volumes between two plans were different from each other in this study, and the value of *GI* only indicated the capability to generate rapid dose-fall off around the target volume [22]. Therefore, the *GI* values indicated that the VMAT could generate more rapid dose-fall off than the tri-Co-60 IMRT; however, the values of V_n%_ of the entire body indicated that the actual normal tissue irradiation was more severe in the VMAT plans than in the tri-Co-60 IMRT plans, as the target volume sizes of the tri-Co-60 IMRT were smaller than those of the VMAT.

The relatively inferior beam delivery system of the ViewRay system appeared to affect the poor plan quality of the target volumes. The large MLC leaf width may impede target conformity and dose homogeneity in the target volume, as several studies previously demonstrated the fine resolution of MLCs could improve the target conformity in addition to dose homogeneity within the target volume [25, 26]. The large penumbrae of the Co-60 sources may also hinder the target conformity. The poor target conformity and homogeneity in the primary and boost plans resulted in almost indistinguishable DVHs between the primary and boost target volumes in the sum plans (Figure 1) [26].

Reviewing the results of irradiation of the body structure excluding the target volumes, the normal tissue irradiations by high doses were larger in the tri-Co-60 IMRT plans than the VMAT plans, while the normal tissue irradiations by intermediate and low doses were larger in the VMAT plans than in the tri-Co-60 IMRT plans. The extensive irradiation by high doses in the tri-Co-60 IMRT appeared to be caused by the large penumbrae of the Co-60 sources, along with MLCs with large leaf width [27]. The 10 MV photon beams of linac could generate more rapid dose fall-off around the target volumes than the Co-60 sources; therefore, the irradiation volume of normal tissue around the target volume by high doses was smaller in the VMAT plans than in the tri-Co-60 IMRT plans. The extensive irradiation of normal tissue by intermediate and low doses of the VMAT may occur because VMAT is an arc therapy technique that is known to induce extensive irradiation of normal tissue by low doses [28]. In addition, the field openings of the VMAT were larger than those of the tri-Co-60 IMRT. This large field openings could result in larger volume of normal tissue irradiation in the VMAT plans than in the tri-Co-60 IMRT plans, consequently, the irradiation of normal tissue by intermediate and low doses could increase in the VMAT plans. With regard to the results of entire body structure including the target volumes, normal tissue irradiation of the tri-Co-60 IMRT plans were consistently lower than those of the VMAT plans. This was also because the target volumes of the VMAT plans were larger than those of the tri-Co-60 IMRT plans.

The limitation of this study is that the tri-Co-60 IMRT plans were not compared to VMAT with small target margins by virtue of rigorous image-guidance. In addition, the target margins in this study were adopted from the literature not from own institutional measurements. Comparing the plan quality of the MR-IGRT to that of VMAT with rigorous IGRT will be performed in the future. Despite of the limitations, this study demonstrated potentials of MR-IGRT for prostate cancer radiotherapy.

## MATERIALS AND METHODS

### Patient selection

After approval from institutional review board, 20 patients with intermediate risk prostate cancer, who received radical VMAT were retrospectively selected for this study. All patients underwent CT scans with a Brilliance CT Big Bore™ (Philips, Cleveland, OH, USA) in the supine position with full bladder. The patient CT images were acquired once before treatment, *i.e.* no CT images were taken during treatment. The slice thickness of the CT image was 1.5 mm. Every patient was immobilized with the Smart Rest™ (Chunsung, Seoul, Republic of Korea), which is a combination of kneefix and feetfix.

### VMAT planning

For each patient, a primary plan was generated to deliver 50.4 Gy (daily dose = 1.8 Gy) to a primary PTV and a boost plan was generated to deliver 30.6 Gy (daily dose = 1.8 Gy) to a boost PTV. The primary CTV was defined by summation of the prostate and seminal vesicle, while the boost CTV was defined based only on the prostate. According to the previous studies, margins for the PTVs were determined as follows [29, 30]. The primary PTV was defined by adding 2 cm margins in every direction from the primary CTV with the exception of the posterior and inferior directions. In the posterior and inferior directions, margins of 1 cm were added to reduce the dose to the rectum. The boost PTV was defined by adding isotropic 0.7 cm margins from the boost CTV. Both primary and boost VMAT plans were generated with the Eclipse™ system (Varian Medical Systems, Palo Alto, CA, USA). Two full arcs and 10 MV photon beams of TrueBeam STx™ (Varian Medical Systems, Palo Alto, CA, USA) were used for the generation of all VMAT plans in this study. The VMAT plans were optimized with the progressive resolution optimizer 3 algorithm (PRO3, ver.10, Varian Medical Systems, Palo Alto, CA, USA) in accordance with the Quantitative Analyses of Normal Tissue Effects in the Clinic (QUANTEC) [21]. After optimization, the anisotropic analytic algorithm (AAA, ver.10, Varian Medical Systems, Palo Alto, CA, USA) was used for dose calculation with a calculation grid of 2 mm. Both primary and boost plans were normalized to cover 95% of the target volume by at least 100% of the prescription dose. The sum plan was generated by summation of the primary and boost VMAT plans.

### Tri-Co-60 IMRT planning

The CT images and the structures used for the generation of the VMAT plans were imported to the treatment planning system (TPS) of the ViewRay system, the MRIdian™ system (ViewRay Inc., Cleveland, OH, USA). No MR images were used for the generation of the tri-Co-60 IMRT plans to eliminate disturbance factors owing to the deformation of the CT images and structures to the MR images. In the same manner as VMAT planning, a primary and a boost tri-Co-60 IMRT plans were generated for each patient. For primary and boost tri-Co-60 IMRT plans, the primary PTV and the boost PTV were generated with 3 mm margins from the primary CTV and boost CTV, respectively, based on the assumption that the margins for daily setup errors and internal organ motions could be minimized with the ViewRay system by virtue of its ART capability. In the same manner as the VMAT planning, prescription doses of 50.4 Gy (daily dose = 1.8 Gy) and 30.6 Gy (daily dose = 1.8 Gy) were delivered to the primary PTV and boost PTV, respectively. A total of 18 fields (7 groups) were used for the generation of both primary and boost plans. Optimization was performed following the QUANTEC guideline [21]. The dose calculation was performed using the Monte Carlo algorithm developed by the manufacturer (ViewRay Inc., Cleveland, OH, USA) with a calculation grid of 3 mm in the presence of a magnetic field. Both primary and boost plans were normalized to cover 95% of the target volume by at least 100% of the prescription dose. The sum plan was also generated by summation of the primary and boost tri-Co-60 IMRT plans.

### Evaluation of treatment plans

From each of the primary and boost plans, D_1%_, D_2%_, D_98%_ and D_99%_ were calculated. The mean dose, minimum dose and maximum doses to the target volumes were also calculated for each of the primary and boost plans. For both primary and boost plans, the *conformity index* (*CI*) and the *HI* were calculated as follows [31].

$$\mathrm{Conformity index}\left(\mathrm{CI}\right)=\frac{\mathrm{Volume of reference isodose}}{\mathrm{Volume of target volume}}$$ (1)

$$\mathrm{Homogeneity index}\left(\mathrm{HI}\right)=\frac{D_{2\%}-D_{98\%}}{\mathrm{mean dose}}$$ (2)

where, the volume of reference isodose = volume irradiated by 100% of the prescription dose.

For both primary and boost plans, the *GI* was calculated as follows [22].

$$\mathrm{Gradient index}\left(\mathrm{GI}\right)=\frac{V_{50\%\mathrm{of the prescription dose}}}{V_{100\%\mathrm{of the prescription dose}}}$$ (3)

From sum plan, D_1%_, D_2%_, D_95%_, D_98%_ and D_99%_ of the primary and boost target volumes were calculated. The mean dose, minimum dose and maximum dose to both primary and boost target volumes were also calculated. The values of *CI* were calculated for both primary and boost target volumes. The *HI* was calculated only for the boost target volume. For bladder, D_55%_, D_30%_, D_25%_, V_80Gy_, V_75Gy_, V_70Gy_, V_65Gy_, and the maximum dose were calculated. For rectum, the values of D_50%_, D_20%_ V_80Gy_, V_75Gy_, V_70Gy_, V_65Gy_, and the maximum dose were calculated. For femoral heads, D_5%_ and the maximum dose to the femoral heads were calculated.

The V_100%_, V_90%_, V_70%_, V_50%_, V_30%_ and V_10%_ of the entire body were calculated. For *Body-PTV*, the aforementioned values were also calculated. The values of *GI*s with 81 Gy (prescribed dose for prostate only) and 50.4 Gy (prescribed dose for seminal vesicle), were calculated.

For each primary and boost plan, the beam-on times were calculated with TPS. With regard to the beam-on time calculation for the tri-Co-60 IMRT plans, the maximum activities of every Co-60 source, which was 15,000 Ci, were assumed [12, 13].

To examine the statistical significance of the differences in the dose-volumetric parameters between the tri-Co-60 IMRT and VMAT plans, the Shapiro-Wilk test was performed to assess whether each data set followed a normal distribution [32]. After normality tests, if both data sets followed normal distributions, a paired *t*-test was performed to calculate *p* values. In the event that the data did not have a normal distribution, the Wilcoxon rank-sum test was performed to calculate *p* values [33, 34]. Differences with *p* values less than 0.05 were regarded as statistically significant in this study.
